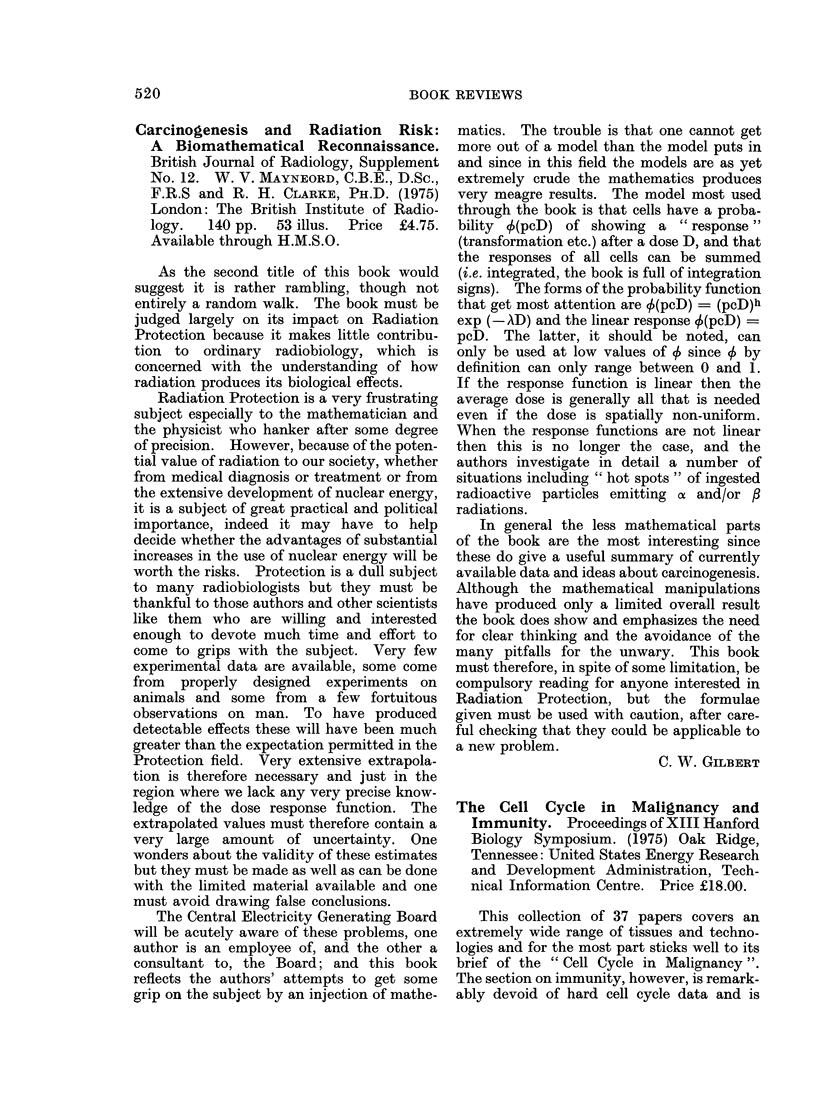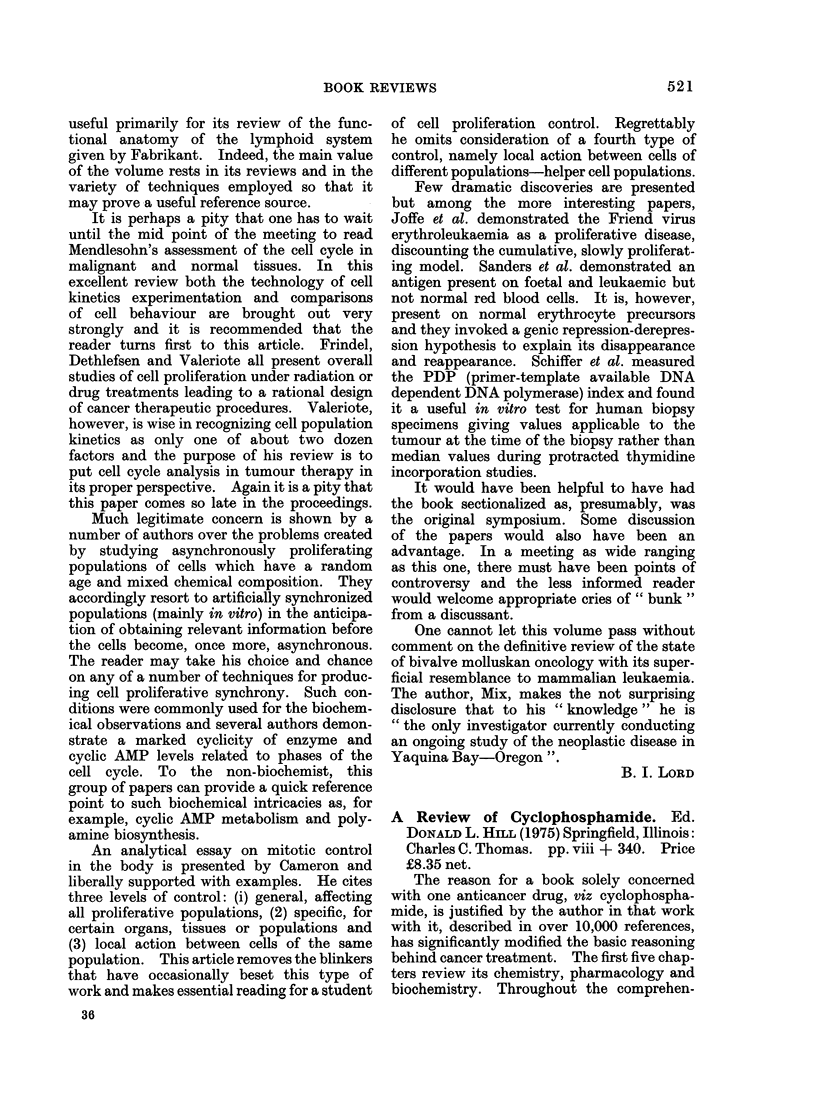# The Cell Cycle in Malignancy and Immunity

**Published:** 1975-10

**Authors:** B. I. Lord


**The Cell Cycle in Malignancy and Immunity.** Proceedings of XIII Hanford Biology Symposium. (1975) Oak Ridge, Tennessee: United States Energy Research and Development Administration, Technical Information Centre. Price £18.00.

This collection of 37 papers covers an extremely wide range of tissues and technologies and for the most part sticks well to its brief of the "Cell Cycle in Malignancy". The section on immunity, however, is remarkably devoid of hard cell cycle data and is useful primarily for its review of the functional anatomy of the lymphoid system given by Fabrikant. Indeed, the main value of the volume rests in its reviews and in the variety of techniques employed so that it may prove a useful reference source.

It is perhaps a pity that one has to wait until the mid point of the meeting to read Mendlesohn's assessment of the cell cycle in malignant and normal tissues. In this excellent review both the technology of cell kinetics experimentation and comparisons of cell behaviour are brought out very strongly and it is recommended that the reader turns first to this article. Frindel, Dethlefsen and Valeriote all present overall studies of cell proliferation under radiation or drug treatments leading to a rational design of cancer therapeutic procedures. Valeriote, however, is wise in recognizing cell population kinetics as only one of about two dozen factors and the purpose of his review is to put cell cycle analysis in tumour therapy in its proper perspective. Again it is a pity that this paper comes so late in the proceedings.

Much legitimate concern is shown by a number of authors over the problems created by studying asynchronously proliferating populations of cells which have a random age and mixed chemical composition. They accordingly resort to artificially synchronized populations (mainly *in vitro*) in the anticipation of obtaining relevant information before the cells become, once more, asynchronous. The reader may take his choice and chance on any of a number of techniques for producing cell proliferative synchrony. Such conditions were commonly used for the biochemical observations and several authors demonstrate a marked cyclicity of enzyme and cyclic AMP levels related to phases of the cell cycle. To the non-biochemist, this group of papers can provide a quick reference point to such biochemical intricacies as, for example, cyclic AMP metabolism and polyamine biosynthesis.

An analytical essay on mitotic control in the body is presented by Cameron and liberally supported with examples. He cites three levels of control: (i) general, affecting all proliferative populations, (2) specific, for certain organs, tissues or populations and (3) local action between cells of the same population. This article removes the blinkers that have occasionally beset this type of work and makes essential reading for a student of cell proliferation control. Regrettably he omits consideration of a fourth type of control, namely local action between cells of different populations—helper cell populations.

Few dramatic discoveries are presented but among the more interesting papers, Joffe *et al.* demonstrated the Friend virus erythroleukaemia as a proliferative disease, discounting the cumulative, slowly proliferating model. Sanders *et al.* demonstrated an antigen present on foetal and leukaemic but not normal red blood cells. It is, however, present on normal erythrocyte precursors and they invoked a genic repression-derepression hypothesis to explain its disappearance and reappearance. Schiffer *et al.* measured the PDP (primer-template available DNA dependent DNA polymerase) index and found it a useful *in vitro* test for human biopsy specimens giving values applicable to the tumour at the time of the biopsy rather than median values during protracted thymidine incorporation studies.

It would have been helpful to have had the book sectionalized as, presumably, was the original symposium. Some discussion of the papers would also have been an advantage. In a meeting as wide ranging as this one, there must have been points of controversy and the less informed reader would welcome appropriate cries of " bunk " from a discussant.

One cannot let this volume pass without comment on the definitive review of the state of bivalve molluskan oncology with its superficial resemblance to mammalian leukaemia. The author, Mix, makes the not surprising disclosure that to his " knowledge " he is " the only investigator currently conducting an ongoing study of the neoplastic disease in Yaquina Bay—Oregon ".

B. I. LORD